# Personalized Bonding Approach for Full‐Mouth Adhesive Rehabilitation in Dentinogenesis Imperfecta

**DOI:** 10.1002/ccr3.70552

**Published:** 2025-06-03

**Authors:** Cyprien Clark, Olivia Kérourédan, Léa Massé

**Affiliations:** ^1^ UFR Dental Sciences – College of Health Sciences University of Bordeaux Bordeaux France; ^2^ Department of Dental Medicine University Hospital of Bordeaux Bordeaux France; ^3^ Department of Restorative Dentistry, UFR Dental Sciences – College of Health Sciences University of Bordeaux Bordeaux France; ^4^ Competence Center for Oro‐Dental Rare Diseases, CCMR O‐Rares University Hospital of Bordeaux Bordeaux France; ^5^ Competence Center for Constitutional Bone Diseases, CCMR MOC University Hospital of Bordeaux Bordeaux France; ^6^ INSERM, BioTis, U1026, University of Bordeaux, Bordeaux, France Bordeaux France; ^7^ Department of Prosthetic Dentistry, UFR Dental Sciences – College of Health Sciences University of Bordeaux Bordeaux France; ^8^ University of Bordeaux, CNRS, MCC, PACEA Pessac France

**Keywords:** adhesion, dentinogenesis imperfecta, minimal invasive dentistry, resin infiltration

## Abstract

Dentinogenesis imperfecta is a rare genetic disorder impacting dentin structure, with an incidence of 1 in 6000 to 1 in 8000 individuals. This condition alters the tooth's color and structure, affecting patients aesthetically, functionally, and socially. Advances in adhesive dentistry offer new therapeutic possibilities, although challenges remain. This article describes the full‐mouth adhesive rehabilitation of a 24‐year‐old male patient diagnosed with DGI, who presented with generalized enamel fractures, shortened crowns, and chronic apical infections. Clinical and radiographic examinations revealed typical features of DGI, including bulbous crowns, short roots, cervical constrictions, and pulpal obliterations. Microscopic analysis of an extracted molar confirmed enamel and dentin structural anomalies, including a smooth dentin‐enamel junction (DEJ) and obliterated dentinal tubules. Given the compromised DEJ and high risk of enamel fracture, a minimally invasive, full adhesive treatment plan was developed. The protocol involved resin infiltration (Icon, DMG) to enhance enamel bonding, followed by the placement of lithium disilicate ceramic crowns in the anterior region and monolithic zirconia bridges posteriorly. Vertical dimension of occlusion was increased using composite abutments and a digital workflow ensured accurate, tissue‐preserving preparations. The treatment significantly improved the patient's quality of life, with the General Oral Health Assessment Index (GOHAI) increasing from 36/60 to 58/60. A one‐year follow‐up showed no prosthetic or biological complications. Further research and long‐term monitoring are necessary to confirm the clinical stability and effectiveness of this approach in managing dentinogenesis imperfecta.

**Summary**

- This case report shows a minimally invasive, full adhesive rehabilitation for managing dentinogenesis imperfecta, involving a pretreatment using resin infiltration for enhancing enamel bond strength.
- The treatment improved quality of life, with stable outcomes at one‐year follow‐up.

## 1 Introduction

Dentinogenesis imperfecta (DGI) is a rare autosomal dominant genetic disorder, affecting approximately 1 in 6000 to 8000 individuals [1]. It is characterized by severe hypomineralization and structural defects in dentin, giving teeth an amber translucency. Dentin, composed primarily of type I collagen and non‐collagenous proteins (DSP, dentin sialoprotein; and DPP, dentin phosphoprotein), is encoded by the DSPP (dentin sialophosphoprotein) gene located on chromosome 4 (4q21.3) [2].

Shields et al. classified DGI into three types (DGI‐I, DGI‐II, DGI‐III) and two forms of dentin dysplasia (DD‐I, DD‐II) [3]. DGI‐I is linked to osteogenesis imperfecta (OI) due to mutations in collagen‐related genes. De La Dure‐Molla later refined this classification, grouping all DSPP‐related conditions under DGI [2]. Molecular studies distinguish between various severities of DGI and dentin dysplasia, with the latter primarily involving root anomalies, while DGI associated with OI is considered syndromic.

Clinically, DGI presents as bluish‐gray or amber translucent teeth with globular crowns, excessive wear, enamel fractures, and spontaneous infections. Radiographs typically show bulbous crowns, shortened roots, and pulpal obliteration, with these features varying widely in severity [2]. The dentin‐enamel junction (DEJ), normally scalloped to absorb stress, appears smooth and wide in DGI, weakening its function and increasing susceptibility to enamel fractures [4, 5]. These structural changes not only affect aesthetics and function but also impact patients' quality of life, often causing discomfort and chewing difficulties [6]. Personalized therapeutic solutions are essential in treatment. Given the compromised DEJ, partial adhesive restorations are not recommended, with peripheral restorations being preferred.7 Recently, infiltrating resin has proven its effectiveness in increasing enamel microhardness as well as adhesion values for restoration [7].

This case is particularly notable for the use of resin infiltration as a pretreatment for full‐mouth adhesive rehabilitation, a rarely described approach in the context of DGI. The combination of structural analysis and the integration of recent adhesive techniques offers new insight into the treatment of such complex cases. This contributes significantly to the limited literature on minimally invasive and fully adhesive management strategies for DGI.

This article presents the case of a 24‐year‐old patient with DGI, illustrating a minimally invasive, fully adhesive rehabilitation using two restorative materials and bonding protocols. Our innovative approach enhances enamel bonding through resin infiltration (Icon DMG), enabling deep penetration of ultra‐fluid resin. A follow‐up on the patient's quality of life was conducted.

## 2 Case History/Examination

A 24‐year‐old male patient was referred to the Bordeaux University hospital for aesthetic and functional management of his oral condition. His medical history was notable only for a pollen allergy, and his general health was excellent. He reported localized pain on the left side (Pain Visual Analogue Scale Score: 4) and dissatisfaction with his smile due to structural anomalies of his teeth, impacting his quality of life with a General Oral Health Assessment Index (GOHAI) [8] score of 36/60. The domains most negatively impacted by his oral health were the functional and psychosocial aspects (Table 1). His main goals were to restore function and improve aesthetics.

**TABLE 1:** GOHAI scores.

In the past 3 month	Never	Seldom	Sometimes	Often	Always
1‐How often did you limit the kinds or amounts of food you eat because of problems with your teeth?	5	4	3	2	1
2‐How often did you have trouble biting or chewing any kinds of food, such as a firm meat or apples?	5	4	3	2	1
3‐How often were you able to swallow comfortably?	1	2	3	4	5
4‐How often have you teeth or dentures prevented you from speaking the way you wanted?	5	4	3	2	1
5‐How often were you able to eat anythingg without feeling discomfort?	1	2	3	4	5
6‐How often did you limit contacts with people because of the condition of your teeth or denture?	5	4	3	2	1
7‐How often were you pleased or happy with the appearance of your teeth, gums or dentures?	1	2	3	4	5
8‐How often did you use medication to relieve pain or discomfort around your mouth?	5	4	3	2	1
9‐How often were you worried or concerned about the problems with your teeth, gums or dentures?	5	4	3	2	1
10‐How often did you feel nervous or self‐conscious because of problems with your teeth, gums or dentures?	5	4	3	2	1
11‐How often did you feel uncomfortable eating in front of people because of problems with your teeth or dentures?	5	4	3	2	1
12‐How often were your teeth or gums sensitive to hot, cold or sweet foods?	5	4	3	2	1

*Note:* The values highlighted in the table are those given by the patient before treatment.

## 3 Methods (Differential Diagnosis, Investigations and Treatment)

**FIGURE 1:**
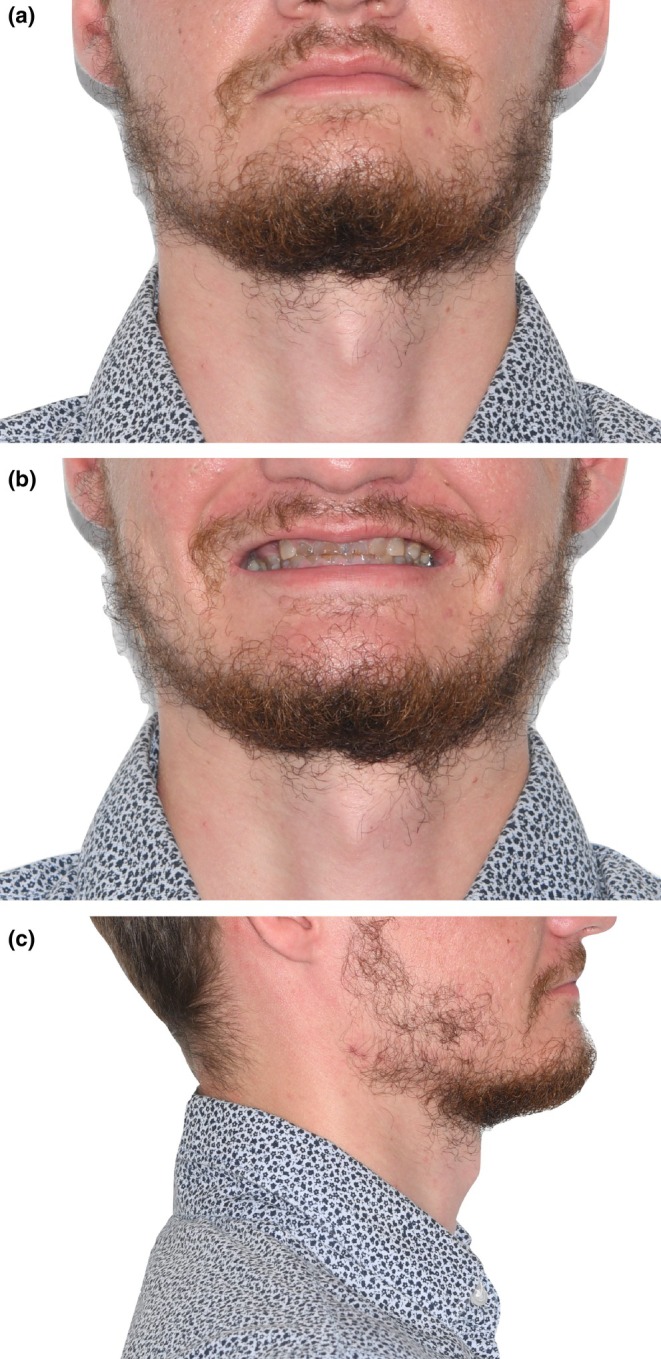
Initial exobuccal examination. (a) Front view. (b) Smile view. (c) Profile view. The hemifaces are symmetrical; the lower facial third is reduced. The nasolabial folds are pronounced; the smile is inverted with aesthetic disharmony.

**FIGURE 2:**
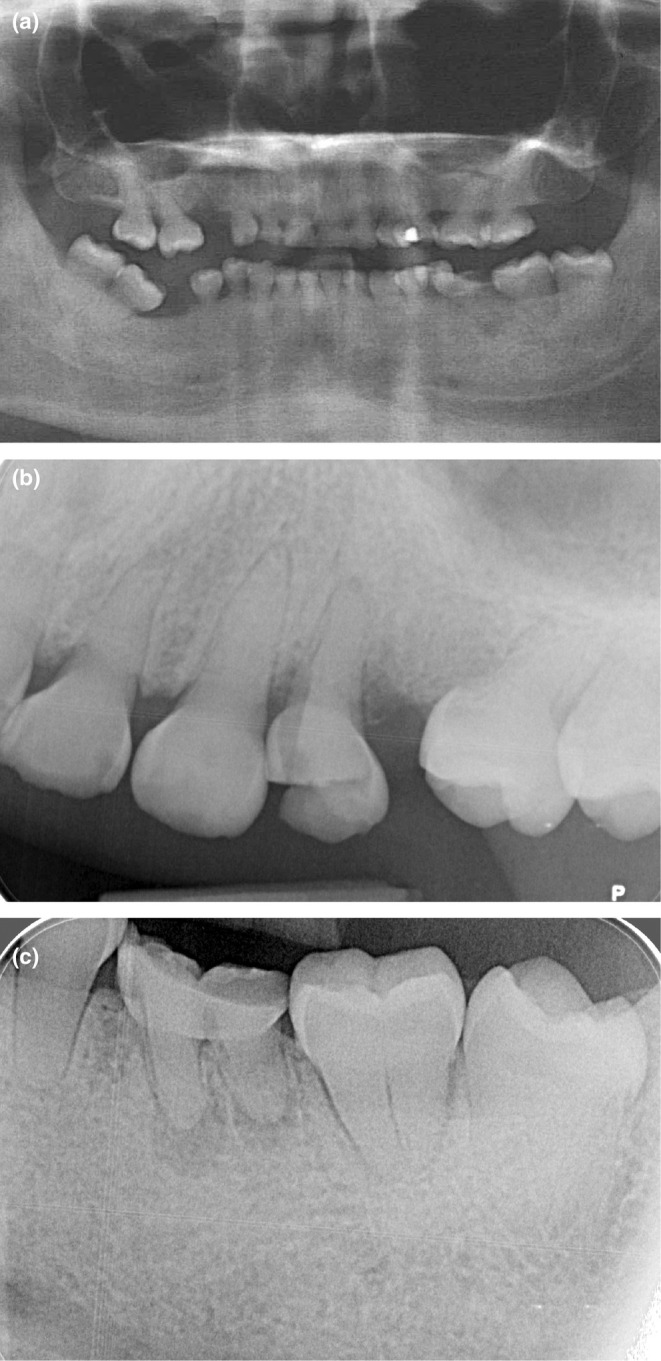
Initial radiographs (a) Initial orthopantomogram, all teeth display distinctive characteristics of DI, with globular crowns, cervical constrictions, and short, thin roots. Pulpal obliteration is observed. (b) Periapical radiograph of sector 2, a radiolucent image is present around the apex of tooth 25. (c) Periapical radiograph of sector 3, tooth 36 exhibits a periapical lesion involving the apices of both roots. Teeth 24 and 36 are to be extracted.

The extraoral examination showed symmetric facial features, reduced lower region, and aesthetic disharmony caused by generalized dyschromia and cervical demineralization (Figure 1). The intraoral exam revealed class 2 canine occlusion, fractures on multiple teeth, and enamel wear due to DGI. Panoramic radiography confirmed the typical DGI presentation, including bulbous crowns, cervical constriction, short and thin roots with pulpal obliteration. Additionally, there was evidence of apical infections in teeth 24 and 36, along with vertical and horizontal bone defects in edentulous regions (Figure 2).

The patient presented with a structural anomaly affecting all teeth, characterized by shortened crowns, enamel fractures, pulpal changes, and bone defects. Chronic apical periodontitis was noted in teeth 24 and 36, along with severe attritional wear and reduced vertical dimension of occlusion. Carious lesions were classified using the SiSta system, with varying scores for multiple teeth. Following this examination, the patient was diagnosed with dentinogenesis imperfecta (DGI) and was advised to consult a geneticist. Genetic testing confirmed a mutation in the DSPP gene (4q21.3), validating the diagnosis of DGI.

**FIGURE 3:**
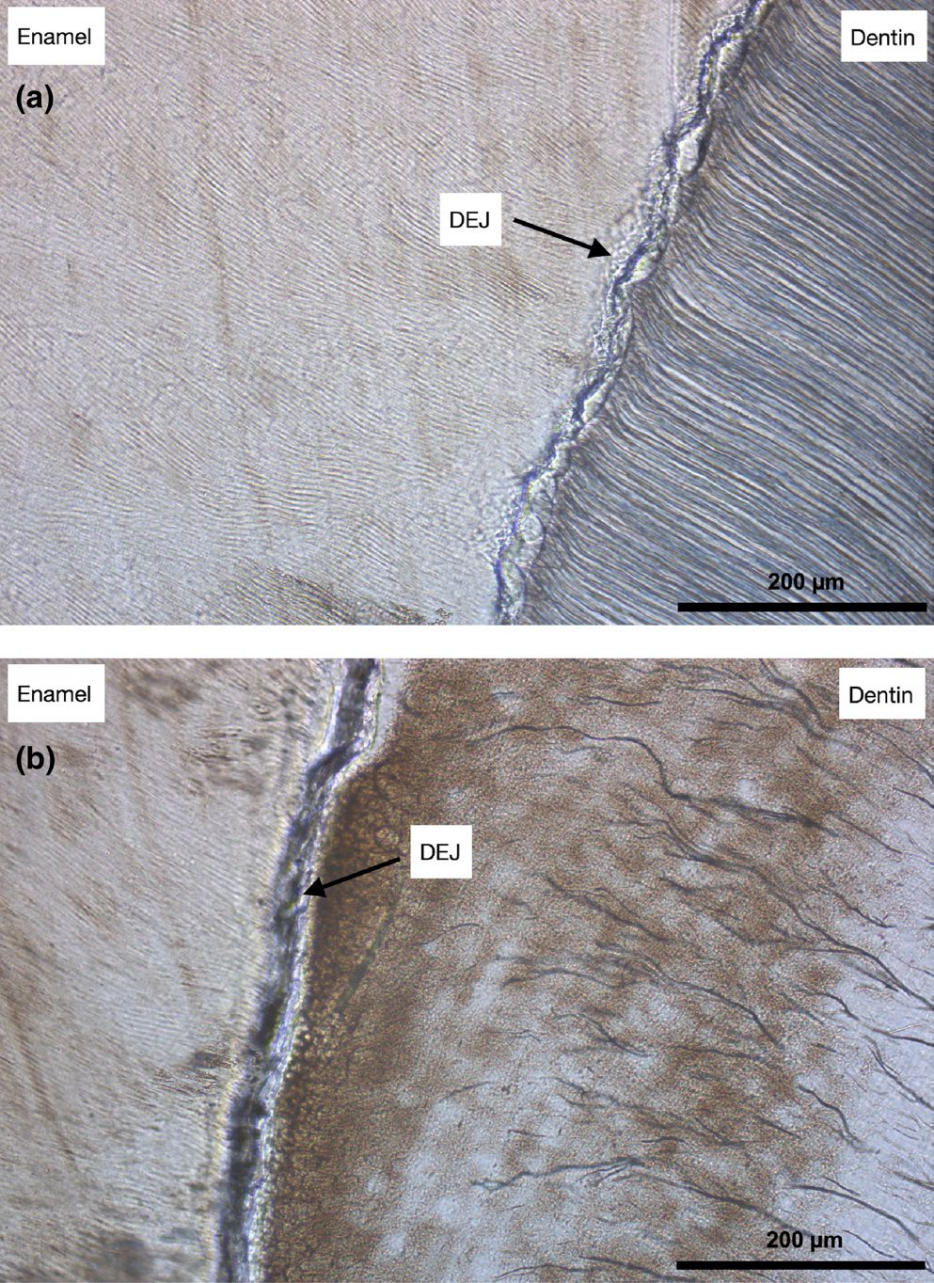
Microscopic analysis. Thin slices, 10 μm thick, were prepared from these sections to allow precise observation of the dental microstructure at a magnification of ×20. (a) The control section belongs to a healthy premolar. (b) Section from the extracted tooth 36, which is affected by dentinogenesis imperfecta.

To confirm structural anomalies and assess the dentin‐enamel junction (DEJ) quality, microscopic analysis was conducted on a vestibulo‐lingual section of tooth 36, which was extracted due to compromised endodontic treatment and an unfavorable crown‐to‐root ratio for preservation, compared with a healthy control. DGI dentin exhibited marked hypomineralization, characterized by considerably fewer dentinal tubules that were obliterated, reduced in size, and arranged in an anarchic, branched manner (Figure 3). In contrast, healthy dentin showed numerous, larger, and well‐aligned tubules. Additionally, cracks were visible in the DGI enamel, giving it an irregular appearance. The DGI DEJ appeared smooth and wide, in contrast to the scalloped DEJ in the control. These findings highlighted the specific structural alterations associated with DGI, underscoring the unique challenges encountered in bonded rehabilitation.

The primary objectives were to restore aesthetics and masticatory function through a minimally invasive approach, preserving as much enamel as possible while ensuring durable bonding. Given the risk of enamel fracture and the compromised DEJ, the treatment plan prioritized peripheral restorations over partial adhesive techniques. The treatment plan was thoughtfully devised in a collegial and multidisciplinary manner. Orthodontic treatment was excluded due to the risk of spontaneous abscesses and the complexity of implant planning. Written informed consent was obtained from the patient before starting treatment and for the publication of this case report, including the use of personal and clinical data.

The treatment strategy involved increasing coronal height by raising the vertical dimension of occlusion (VDO) and employing full zirconia bridges posteriorly for strength. For the anterior regions, minimally invasive CAD‐fabricated crowns in lithium disilicate‐reinforced ceramics were chosen. Resin infiltration using the Icon (DMG) protocol was applied to optimize enamel bonding.

**FIGURE 4:**
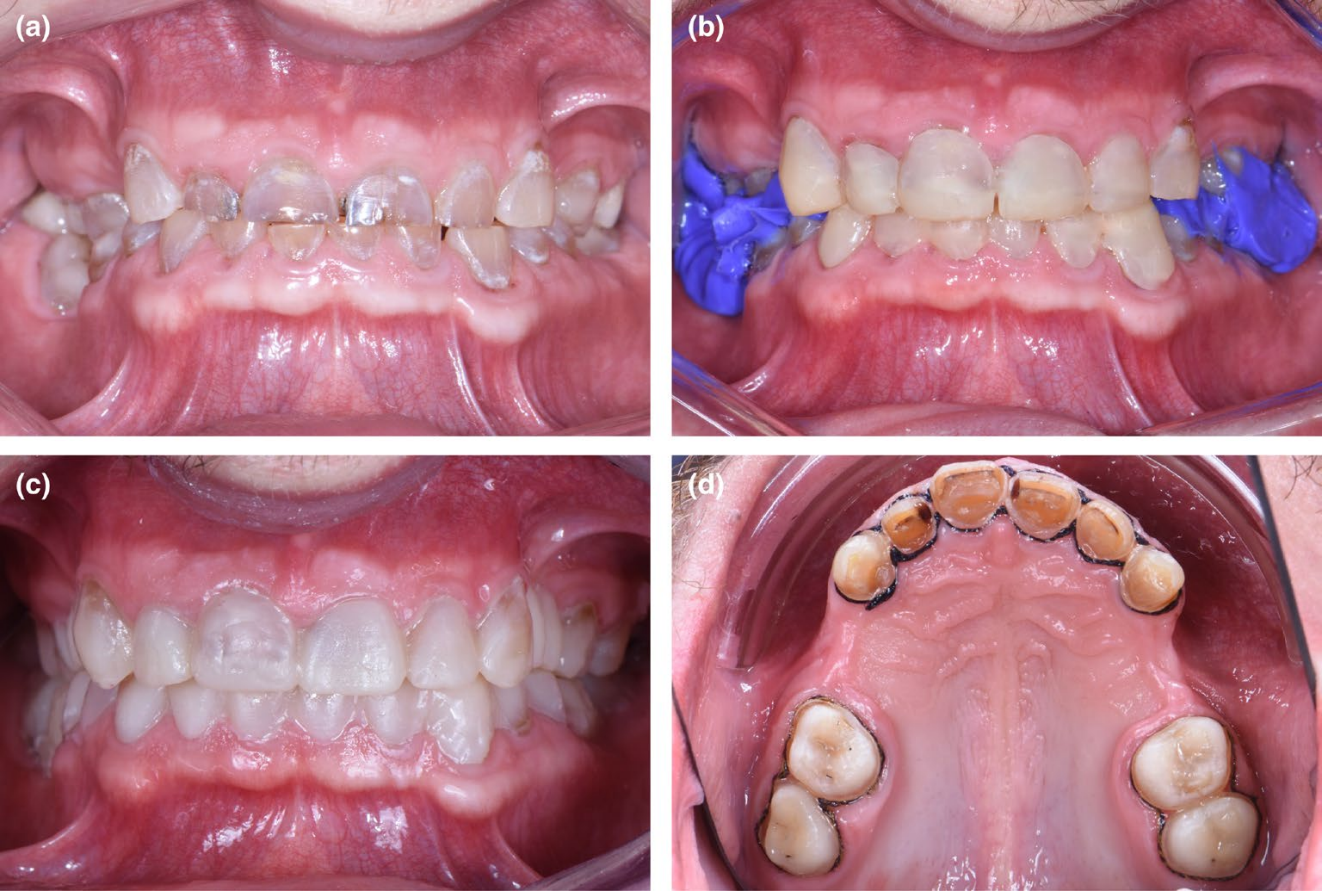
Design of the Comprehensive Prosthetic Plan and calibrated tooth preparation. (a) Initial situation in an intraoral view. (b) Instant mock‐up using the “Direct Free‐Hand Composite Up” technique : layers of composite are applied from canine to canine to establish the new occlusal vertical dimension (OVD) based on aesthetic principles. (c) After a complete wax‐up is performed, the project is validated intraorally using the technique of templates or full mock‐ups. (d) calibrated tooth preparation of the maxillary to maximize enamel preservation.

To establish the prosthetic plan, initial impressions were taken to produce study models, followed by an anterior mock‐up using the “Direct Free‐Hand Composite Up” technique [9] to adjust vertical dimension of occlusion (VDO) (Phase 1). Composite abutments were used to maintain the new VDO, recorded with a facebow for accurate maxillary positioning. A wax‐up simulated final restorations, validated intraorally using the full mock‐up technique (ATP) [10]. The patient retained the mock‐up for 1 month with weekly adjustments, ensuring comfort and adapting the final treatment plan accordingly (Figure 4).

**FIGURE 5:**
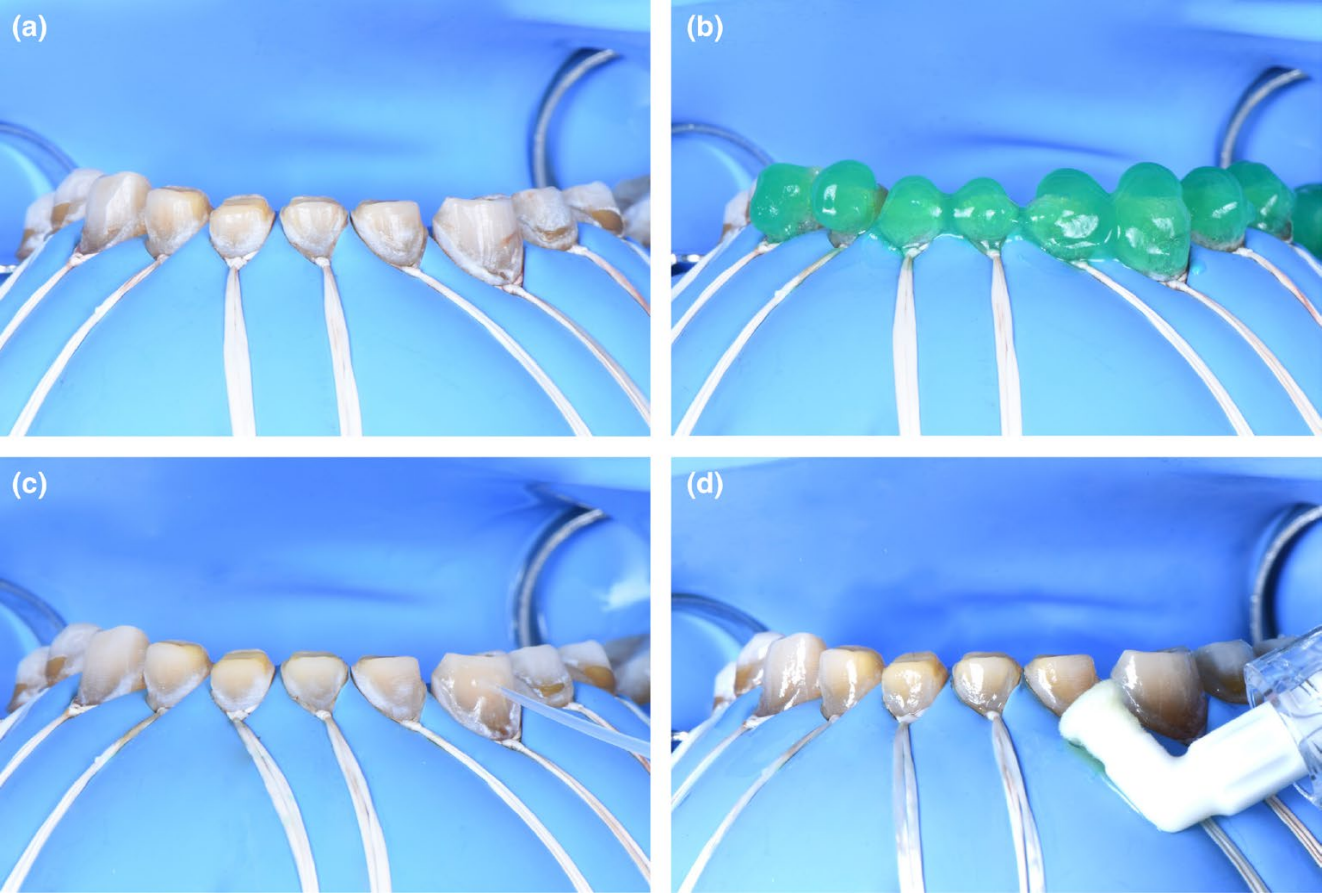
Erosion‐infiltration protocol, optimization of affected enamel prior to bonding. (a) strict isolation (rubber dam, clamps, ligatures). (b) IconEtch (DMG, Pred Laboratory): 15% HCl etching for 2 min. (c) IconDry (DMG, Pred Laboratory): Drying with 99% ethanol for 30 s. (d) IconInfiltrant (DMG, Pred Laboratory): Resin infiltration (TEGDMA) over the entire enamel surface for 3 min. After a 40‐s light curing, a second infiltration for 1 min is performed, followed by another 40‐s light curing.

**FIGURE 6:**
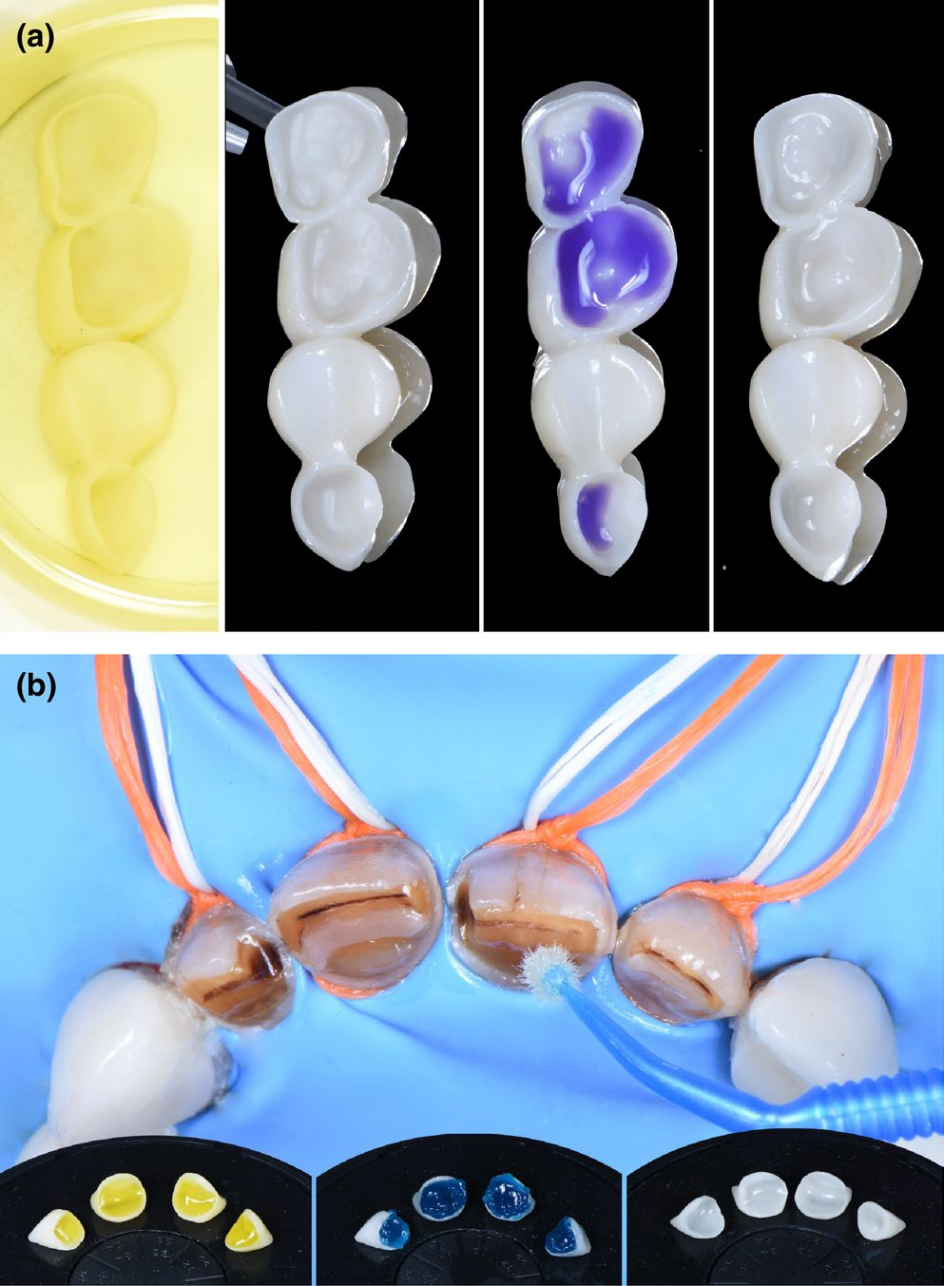
Preparation of zirconia bridges and anterior prosthetic components in lithium disilicate. (a) Preparation of the prosthetic intrados: From left to right, disinfection and degreasing with alcohol, sandblasting, cleaner application, prosthetic primer application. (b) Hydrofluoric acid for 20 s, treatment of over‐etched areas with orthophosphoric acid for 120 s, silanization for 60 s, application of universal adhesive on all bonding surfaces, assembly using a composite without adhesive potential.

Phase 2 involved prosthetic treatment. Digital impressions were taken with the mock‐up in place, allowing calibrated tooth preparation to maximize enamel preservation (Figure 4) [11]. Bridges were designed with adequate thickness and connectors based on location. Try‐in waxes validated the fit and occlusion, and the final prosthetics were fabricated in the lab using monolithic ceramic. Prior to bonding, resin infiltration (Icon (DMG) protocol) was performed on enamel to optimize adhesion (Figure 5). Two distinct bonding protocols were applied for the zirconia bridges [12, 13, 14] and glass ceramic crowns [15] to maximize bond strength and durability (Figure 6). Following bonding, radiographic verification confirmed no excess adhesive material remained.

## 4 Conclusion and Results (Outcome and Follow‐Up)

**FIGURE 7:**
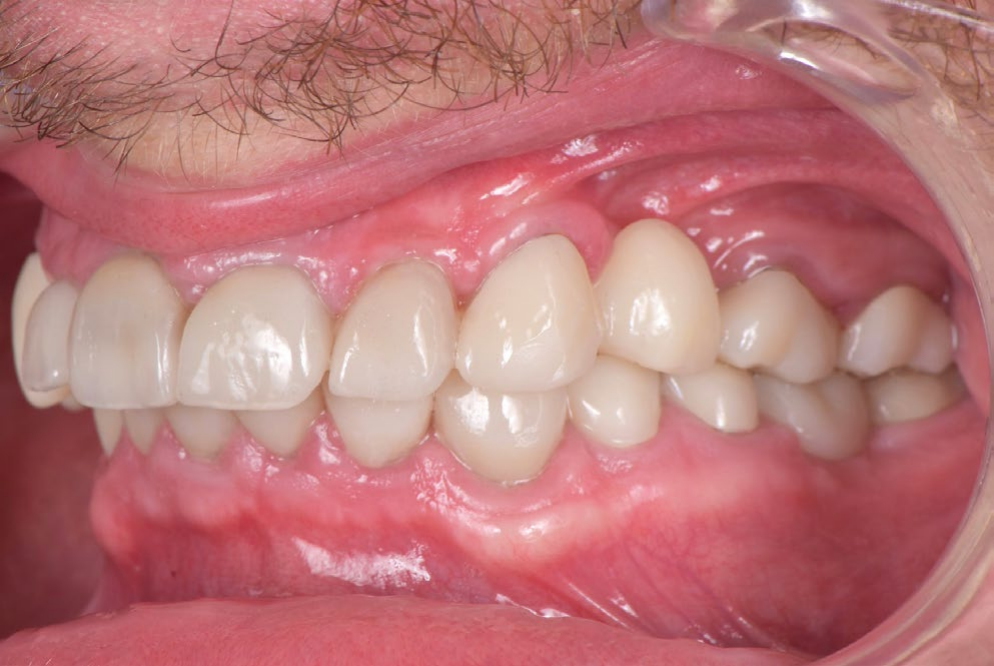
Situation 2 weeks post‐bonding. Left lateral view.

**FIGURE 8:**
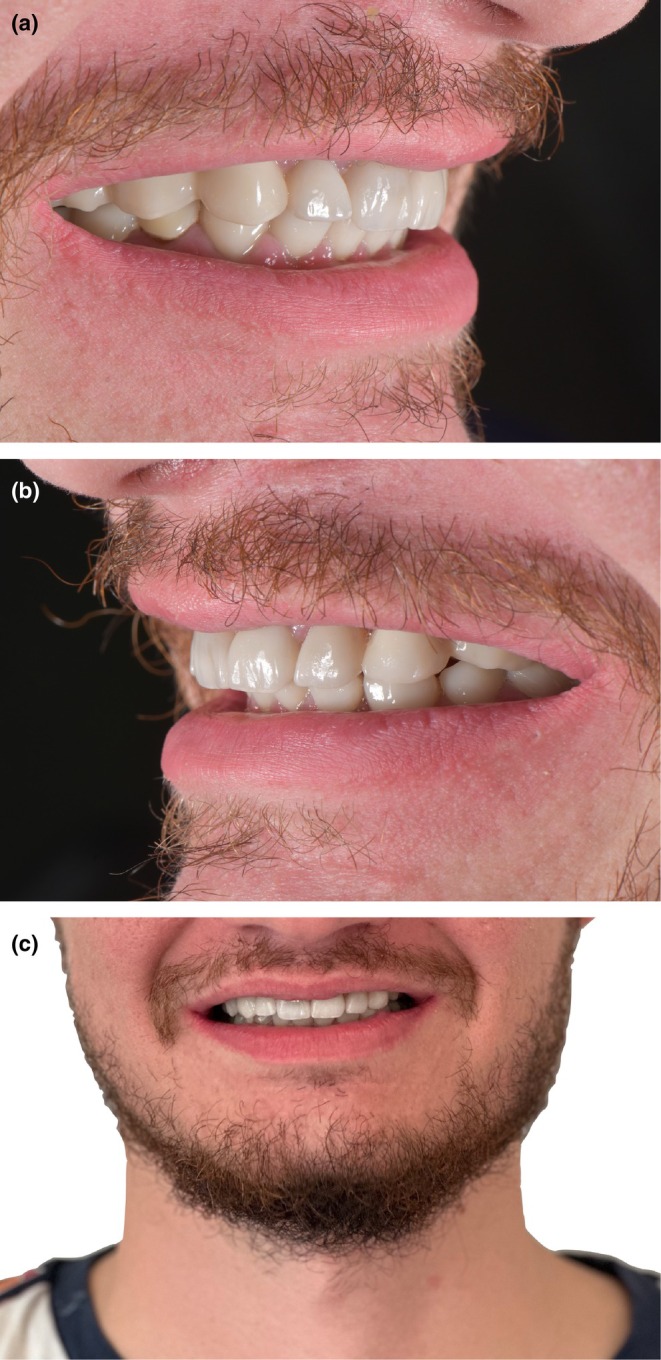
Extraoral view at 13 months post‐bonding. (a, b) Lateral views. (c), Frontal view.

At a two‐week post‐operative check, the patient expressed high satisfaction with the aesthetic outcome (Figure 7). Thirteen months post‐treatment, he reported significant functional and aesthetic improvements, with a marked enhancement in quality of life (Figure 8). The GOHAI score increased from 36/60 to 58/60, indicating improved oral health and psychosocial well‐being (Table 2).

**TABLE 2:** GOHAI scores.

In the past 3 month	Never	Seldom	Sometimes	Often	Always
1‐How often did you limit the kinds or amounts of food you eat because of problems with your teeth?	5	4	3	2	1
2‐How often did you have trouble biting or chewing any kinds of food, such as a firm meat or apples?	5	4	3	2	1
3‐How often were you able to swallow comfortably?	1	2	3	4	5
4‐How often have you teeth or dentures prevented you from speaking the way you wanted?	5	4	3	2	1
5‐How often were you able to eat anythingg without feeling discomfort?	1	2	3	4	5
6‐How often did you limit contacts with people because of the condition of your teeth or denture?	5	4	3	2	1
7‐How often were you pleased or happy with the appearance of your teeth, gums or dentures?	1	2	3	4	5
8‐How often did you use medication to relieve pain or discomfort around your mouth?	5	4	3	2	1
9‐How often were you worried or concerned about the problems with your teeth, gums or dentures?	5	4	3	2	1
10‐How often did you feel nervous or self‐conscious because of problems with your teeth, gums or dentures?	5	4	3	2	1
11‐How often did you feel uncomfortable eating in front of people because of problems with your teeth or dentures?	5	4	3	2	1
12‐How often were your teeth or gums sensitive to hot, cold or sweet foods?	5	4	3	2	1

*Note:* The values highlighted in the table are those given by the patient after treatment.

This case demonstrates that a personalized bonding approach incorporating resin infiltration can effectively address the structural challenges of DGI, leading to stable aesthetic and functional outcomes. The integration of microscopic analysis with advanced adhesive protocols supports a minimally invasive strategy that preserves dental tissues. These findings highlight the potential of this approach as a conservative alternative in managing structurally compromised dentitions and underscore the need for further clinical research.

## 5 Discussion

This case highlights the challenges of managing DGI through prosthetic rehabilitation, especially with bonding efficacy. Microscopic analysis showed significant enamel and dentin alterations that limit conventional bonding. Innovative solutions like Icon (DMG) resin infiltration and 10‐MDP primers optimize enamel cohesion, while minimally invasive crowns and 360° veneers ensure enamel preservation, functionality, and aesthetics.

While the literature on DGI‐related prosthetic treatments is limited, prior studies have explored both implant‐based approaches and full‐coverage crowns, often requiring significant tooth reduction and potentially exacerbating enamel and dentin fragility [16, 17]. Etienne et al. [18] advocate for more conservative approaches, similar to those recommended for tooth wear, but with covering and peripheral preparation forms due to enamel and DEJ defects. The fully adhesive and minimally invasive approach in this case adheres to the therapeutic gradient, balancing structural preservation with aesthetic and functional outcomes. The choice of monolithic ceramics, especially zirconia for posterior restorations, offers high survival rates and resistance to fracture, suitable for high‐stress areas, as demonstrated in the literature [19, 20]. Our findings are consistent with reports of enamel alterations, including cracks and irregularities, in DGI cases [21, 22, 23, 24], and they underscore the potential of resin infiltration to reinforce weakened enamel and optimize bonding.

The therapeutic compromises, including skipping prior orthodontic alignment and minimal gingival adjustments, slightly affected occlusal and aesthetic outcomes but did not significantly impact overall results. Continued follow‐up and longitudinal studies are needed to confirm the long‐term stability of this approach, including resin infiltration‐based pretreatment, but initial outcomes show promising improvements in patient quality of life and treatment durability.

Follow‐up studies are needed to validate the long‐term stability of this resin‐infiltration‐based approach, but initial outcomes suggest enhanced patient quality of life and durable results.

## Author Contributions

**Cyprien Clark:** resources, validation, visualization, writing – original draft, writing – review and editing. **Olivia Kérourédan:** conceptualization, data curation, methodology, writing – review and editing. **Léa Massé:** conceptualization, data curation, methodology, supervision, writing – original draft, writing – review and editing.

## Ethics Statement

The publication of this case report was approved by the Bordeaux University Hospital Research Ethics Committee (CER‐BDX 2024‐324).

## Consent

Written informed consent was obtained from the patient for the publication of this case report, including the use of personal and clinical data in accordance with the journal's patient consent policy.

## Conflicts of Interest

The authors declare no conflicts of interest.

## Data Availability

Research data are not shared.
